# Chronic inflammatory demyelinating polyneuropathy following COVID-19 vaccination: a case report and literature review

**DOI:** 10.1186/s12883-024-03756-3

**Published:** 2024-07-29

**Authors:** Mohammad Ali Bahramy, Zahra Hashempour, Reza Shahriarirad

**Affiliations:** 1 Department of Neurology, Naft Grand Hospital, Ahvaz, Iran; 2 School of Medicine, Shiraz University of Medical Sciences, Shiraz, Iran; 3 Thoracic and Vascular Surgery Research Center, Shiraz University of Medical Sciences, Shiraz, Iran

**Keywords:** Case report, Chronic Inflammatory Demyelinating, COVID-19 vaccine, Polyradiculoneuropathy

## Abstract

**Background:**

Severe post-vaccination neurological complications are rare. Chronic Inflammatory Demyelinating Polyneuropathy (CIDP) is an immune-mediated polyneuropathy affecting the peripheral nerve roots, which is not well described as a post-vaccination side effect. Here, we present a rare complication of vaccination against SARS-CoV-2, reaching a diagnosis of CIDP.

**Case presentation:**

A 67-year-old diabetic male presented with lower extremity paresthesia and weakness following the third dose of the Sinopharm (BBIBP-CorV) vaccine. Despite initial dismissal as a diabetic complication, symptoms escalated, affecting all extremities. Electromyography study revealed abnormal spontaneous activity with chronic reinnervation changes, which was more significant in the lower extremities. Based on the clinical course, radiographic imaging, and laboratory data, a diagnosis of CIDP with severe axonal demyelinating features was established. Treatment with intravenous immunoglobulin (IVIg), prednisolone, and azathioprine resulted in marked improvement of the upper extremities but limited recovery in distal lower extremity muscles.

**Conclusion:**

Although CIDP is a rare complication following COVID-19 vaccination, it should be considered in the differential diagnosis. Timely diagnosis of vaccine-induced CIDP is challenging, and any delay can adversely affect treatment response in affected patients.

**Supplementary Information:**

The online version contains supplementary material available at 10.1186/s12883-024-03756-3.

## Background

Vaccines are the most promising way of combating the Coronavirus disease 2019 (COVID-19) pandemic [1]. Sinopharm vaccine, also known as the BBIBP-CorV vaccine, is one of the several candidates for COVID-19 vaccination [2]. The rapid creation of vaccines has increased the potential vaccine safety hazards. Aside from the vaccines’ mild and common side effects, some rare, serious adverse reactions, such as anaphylaxis, thrombotic thrombocytopenia, myopericarditis, and Guillain-Barré syndrome (GBS), are increasingly reported [1]. Acute transverse myelitis, acute disseminated encephalomyelitis, and acute demyelinating polyneuropathy are other unexpected neurological adverse events [3].

Chronic inflammatory demyelinating polyradiculoneuropathy (CIDP) is a rare, treatable immune-mediated neuropathy, typically manifesting as a symmetric progressive or relapsing sensorimotor polyneuropathy affecting all extremities. CIDP has a challenging diagnosis and treatment since the clinical presentation is diverse and accurate biomarkers are lacking [4]. The process of axonal loss in CIDP worsens with time and adversely affects treatment response. This makes underdiagnoses and diagnostic delays highly undesirable and can cause significant impairment of quality of life [5].

Recent studies have raised the possibility of a connection between COVID-19 vaccination and CIDP. Here, we report a case of CIDP presenting with progressive symptoms of demyelinating neuropathy following the third dose of the Sinopharm vaccine. We believe that our report will contribute to the body of evidence regarding post-vaccination complications, and increase awareness among clinicians to make more informed decisions regarding patients at risk for these adverse events [6–8]. This case is reported in adherence to CARE guidelines.

## Case presentation

A 67-year-old male presented with a progressive tingling sensation and paresthesia in both lower extremities. Seven days before the onset of symptoms, the patient received a third dose of the Sinopharm vaccine against SARS-CoV-2. He is a known case of knee arthrosis and diabetes mellitus, and his blood sugar was controlled with metformin. He was a former boxing teacher, physically fit, and weighted 85 kg. He also denied any history of recent infection.

At first, the paresthesia was neglected since the patient assumed it was a possible complication of diabetes. However, the ascending progression, followed by increasing weakness of the lower extremities to the extent that he had trouble initiating ambulation, compelled the patient to visit an orthopedic specialist. A lumbosacral magnetic resonance imaging (MRI) was requested, which revealed mild bulging of the L5-S1 intervertebral disc. Initial treatment with gabapentin 300mg twice a day (for paresthesia) and physical therapy was started. However, it did not yield any improvement, and within two months, the paresthesia and weakness extended to the upper extremities. Subsequently, five months following the initiation of symptoms, he was referred to our center for further evaluation.

On neurological examination, all cranial nerves were intact. Strength testing revealed normal strength of cervical muscles (on the Medical Research Council (MRC) scale; grade 5/5) but reduced force in all four extremities, especially in the deltoid muscle (grade 4/5) and lower extremities (proximal grade 3/5, distal grade 2/5). Additionally, wrist and finger extension-flexion power were diminished (grade 4/5), as were ankle dorsiflexion and plantar flexion (grade 2/5). Sensory testing showed an impaired sense of position and vibration and reduced pin-prick sensation in both the upper and distal lower extremities. On examination, the patient had action and postural tremor in his upper extremities, along with gait ataxia and generalized areflexia. The patient showed no sign of autonomic dysfunction, dysphagia, or incontinence.

He underwent electromyography and nerve conduction studies (EMG-NCV) and sensory nerve action potential (SNAP) in all four limbs, which revealed absent compound muscle action potentials (CMAP) amplitudes in lower limbs (tibial and peroneal nerves). Median, ulnar, and radial nerve conduction studies revealed increased distal latency and significant amplitude reduction, with significantly slowed conduction velocity and conduction block. It is worth noting that, due to the demyelination features of NCSs, conduction delay was highly remarkable (Table 1). Electromyography study revealed abnormal spontaneous activity with chronic reinnervation changes, which was more significant in the lower extremities (Table 2). These findings were in favor of chronic inflammatory demyelinating polyneuropathy (CIDP) with secondary axonal degeneration.

Due to the clinical course of the disease and considering the results of EMG-NCV, the patient underwent cerebrospinal fluid (CSF) analysis. As demonstrated in Table 3, results of CSF analysis showed elevated protein levels (protein: 210 mg/dL, normal < 45 mg/dL) without pleocytosis (WBC count: 4). Cervical MRI, vasculitis and paraneoplastic panel, and serum and urine protein electrophoresis were unremarkable.

Laboratory data including blood cell counts, basic metabolic panel, renal function test, liver function tests, thyroid function tests, vitamin B12, folate, copper, calcium, vitamin D3, hemoglobin A1C, total protein, albumin, erythrocyte sedimentation rate, C-reactive protein, urine analysis, serum protein electrophoresis, and urine protein electrophoresis were all within normal limits. Antinuclear antibody, serum perinuclear staining antineutrophil cytoplasmic antibody (P-ANCA), cytoplasmic-ANCA, and ganglioside antibody tests were negative. The human immunodeficiency virus, hepatitis B, and hepatitis C tests were also negative.

**Table 1:** Motor nerve conduction study in a 67-year-old male with preliminary diagnosis of CIDP

Nerve/site	Latency (ms)			Amplitude (mV)			Distal Distance (cm)	Velocity (m/s)		
	On admission	After treatment	Reference	On admission	After treatment	Reference		On admission	After treatment	Reference
Median Left (Abductor pollicis brevis)										
Wrist	8.3	8.1	≤4.4	0.75	0.93	≥4	7	20.4	22.1	≥49
Elbow	18.1	17.8		0.34	0.51					
Median Right (Abductor pollicis brevis)										
Wrist	7.8	7.4	≤4.4	1.9	2.2	≥4	7	21.3	23.4	≥49
Elbow	17.7	16.3		0.8	1.1					
Ulnar Left (Abductor digiti minimi)										
Wrist	5.3	5.2	≤3.3	3.7	3.6	≥6	7	18.5	20.6	≥49
Elbow	16.1	15.5		1.3	1.3					
Ulnar Right (Abductor digiti minimi)										
Wrist	5.1	4.7	≤3.3	2.9	3.2	≥6	7	19.1	23.2	≥49
Elbow	16.2	16.1		1.1	1.2					
Radial Left (Extensor indicis proprius)										
Forearm	8.2	7.9	≤2.9	0.56	0.52	≥2	5	19.1	22.5	≥49
Elbow	11.4	11.2		0.21	0.20					

*Abbreviations*: *CIDP* Chronic inflammatory demyelinating polyneuropathy

**Table 2:** EMG Findings in a 67-year-old male with preliminary diagnosis of CIDP

Muscle	Side	Ins. Act	Fibs	Pos. Wave	Fasc	Polyphasia	Amplitude	Duration	Recruitment
Biceps Brachii	Left	Normal	0	0	0	0	Normal	Normal	Normal
	Right	Normal	0	0	0	0	Normal	Normal	Normal
Extensor Carpi Radialis	Right	Normal	0	0	0	0	Normal	Normal	Normal
Extensor Digitorum Communis	Left	Normal	0	0	0	0	Normal	Normal	Normal
Flexor Carpi Radialis	Left	Normal	0	0	0	0	Normal	Normal	Normal
	Right	Normal	0	0	0	0	Normal	Normal	Normal
1st Dorsal Interosseous	Left	1 +	1 +	1 +	0	+	+ 1	Normal	Reduced
	Right	1 +	1 +	1 +	0	+	+ 1	Normal	Reduced
Quadriceps	Left	2 +	2 +	2 +	0	+ +	+ 2	Normal	Reduced
	Right	2 +	2 +	2 +	0	+ +	+ 2	Normal	Reduced
Peroneus Longus	Left	2 +	2 +	2 +	0	+ +	+ 2	Increased	Reduced
	Right	2 +	2 +	2 +	0	+ +	+ 2	Increased	Reduced
Tibialis Anterior	Left	2 +	2 +	2 +	0	+ + +	+ 2	Increased	Reduced
	Right	2 +	2 +	2 +	0	+ + +	+ 2	Increased	Reduced

*Abbreviations*: *CIDP* Chronic inflammatory demyelinating polyneuropathy, *EMG* Electromyography, *Ins. Act.* Insertional activity, *Fasc*. Fasciculation, *Fibs*. Fibrillation potentials, *Pos. Wave* Positive sharp wave

**Table 3:** Results of cerebrospinal fluid analysis in a 67-year-old male with paresthesia and weakness

CSF parameters	On admission	After treatment	Normal values
Glucose	65	57	40–70
Protein (mg/dL)	210	60	15–45
WBC count	4 Lymphocytes, No PMN	2 Lymphocytes, No PMN	0–5 cells
RBC count	Absent	Absent	Absent
Xanthochromia	Absent	Absent	Absent

*Abbreviations: CSF* Cerebrospinal fluid, *PMN* Polymorphonuclear neutrophil, *RBC* Red blood cell, *WBC* White blood cell

Based on the clinical presentation and EMG-NCV results, we ruled out other possible differential diagnoses, including diabetic neuropathy, diabetic amyotrophy, and other immune-mediated neuropathies. The patient was admitted with the impression of severe CIDP with secondary axonal degeneration and was treated with an initial dose of intravenous immunoglobulin (IVIg) 170 gr (2 gr/kg, weight: 85kg), along with continuing physiotherapy sessions. Following no significant improvement in the first month of treatment, a second round of treatment was started with prednisolone 50mg daily, IVIg 85 gr (1 gr/kg, 3 doses), and Azathioprine 50 mg daily, which was then advanced to 50 mg twice a day.

Following treatment with IVIg, prednisolone, and Azathioprine, the patient reported gradual, marked improvement and regained normal function of the upper extremities (grade 5/5) and the ability to stand up without assistance. Examination confirmed improved motor strength in the proximal muscles of both lower extremities but no significant improvement in the distal lower extremity muscles (grade 5/5 in the proximal and 2/5 in the distal muscles).

To assess the extent of recovery, a second CSF analysis was performed, which displayed a significant decrease in the protein level (60 mg/dL). Also, NCV findings at the 3-month follow-up showed no significant change except for the absence of spontaneous activity (fibrillation and positive sharp wave). He was discharged with ongoing IVIg treatment and a tapering dose of prednisolone, along with a routine neurology clinic follow-up.

## Discussion and conclusions

Neuropathies after vaccination are rare and poorly understood events. About 1.5% of CIDP patients have a history of antecedent vaccination, which is distinctly unusual [9]. CIDP can be a challenging diagnosis due to the heterogeneity of presentations, ranging from distal versus proximal onset, symmetric versus asymmetric, and sensory versus motor variants. A slow disease onset may prevent rapid recognition and delay treatment [10]. In this study, we presented a rare complication of the BBIBP-CorV vaccine in a 67-year-old man who presented with symmetrical polyneuropathy of four limbs involvement. We further performed a literature review for all COVID-19 vaccine-induced CIDP patients, in which our findings are reported in Table 4.

**Table 4:** Literature review of characteristics of newly-diagnosed CIDP patients as a complication of COVID-19 vaccination

Authors	Age/ Sex	Comorbidity	Type of Vaccine/dose	Symptoms/Signs	Days from vaccine	CSF	EMG-NCV	Imaging	Treatment
**Oo et al. (2021) **[11]	72/M	Idiopathic neuropathy, influenza	AstraZeneca/first Influenza	• Progressive ascending lower limb sensory motor involvement • Predominantly proximal quadriparesis • Sensory changes	21 42	• Protein: 0.55 g/L • WBC: 0/mm^3^	• Absent sensory responses • Prolonged distal motor latency with reduced nerve conduction velocity	• NA	• IVIg • Rehabilitation
**Suri et al. (2021) **[10]	47/F	DM, hypertension, and COVID-19 (7 months ago)	AstraZeneca/ first	• Progressive pure motor-flaccid quadriparesis, facial weakness • Areflexia • Recurrence with cranial nerve involvement (sixth and seventh)	17	• Protein: 250 mg/dL • Cells: 0/mm^3^	• Demyelinating polyradiculoneuropathy • Abnormal facial and blink reflexes	• Brain MRI: hyperintensities in white matter • Lumber MRI: enhancing cauda equina thickening, avid lesion in fused PETMRI	• IVIg • Oral prednisolone • Azathioprine
**Bagella et al. (2021**) [9]	47/F	NA	Astra Zeneca/first	• Asymmetric bilateral facial weakness • Paresthesia in the tongue and face • Lower limbs areflexia • Sensory ataxia • Wide-based gait	16	• Protein: 110 mg/dL • WBC: 0/mm^3^ • No intrathecal IgG	• Slowing of conduction velocities • Prolonged distal latency and conduction blocks • Absent F response	• Brain and spinal MRI: enhancement of the facial nerves, lower thoracic nerve roots and cauda equina	• Initial IVIg followed by maintaining IVIg therapy every 6 months
**Wen et al. (2022) **[12]	23/M	None	Inactivated coronavirus vaccine/second	• Left upper limb weakness (progression to numbness and weakness of all extremities) • Areflexia	1	• Protein: 0.99 g/L • Cells: 4×10^6^/L	• Reduced SNAP amplitudes in upper limbs • Reduced CMAP amplitude in all extremities • Prolonged tibial F wave latencies • EMG: neurogenic damage	• Normal brain and spinal MRI	• IVIg • High dose intravenous glucocorticoid • Plasmapheresis • Rituximab
**De Souza et al. (2022) **[13]	51/M	Congenital deafness, coronary artery disease	AstraZeneca/ first	• Low back pain • Lower limb and bifacial weakness • Severe areflexic quadriparesis • Sensory loss in the feet	14	• Protein: 0.70 g/L • WBC: 1 lymphocyte	• Prolonged distal latencies • Reduced NCV	• NA	• IVIg • Plasma exchange • Rehabilitation
	72/M	Localized prostate cancer, type 2 DM, hypertension, hypothyroidism	AstraZeneca/ first	• Bilateral lower limb paresthesia • Dysarthria • Gait dysfunction	21	• Protein: 2.02 g/L without pleocytosis	• Mixed axonal and demyelinating polyradiculoneuropathy	• Spinal MRI: enhancement of cauda equina and surface of the lower dorsal spinal cord	• IVIg • Low-dose prednisolone
	72/M	Hypertension	AstraZeneca/ first	• Paresthesia in hands and feet • Lower limb weakness • Difficulty ambulating • Bifacial weakness • Right abducent nerve palsy • Dysarthria • Dysphagia • Areflexia	14	• Protein: 1.964 g/L • WBC: 9 lymphocytes	• Prolonged distal latencies • Reduced NCV • Conduction block in all of the limbs	• Spinal MRI: thickening and enhancement of lumbosacral nerve roots	• IVIg
	72/M	Demyelinating neuropathy (stable without treatment for three years)	AstraZeneca/ first	• Paresthesia in distal lower limbs • Proximal weakness of all limbs • Areflexia and hypoalgesia in the hands, legs, and feet • Impaired proprioception • Sensory ataxia	21	• Protein: 0.55 g/L • Acellular	• Prolonged distal latencies • Reduced NCV • Temporal dispersion in lower limbs	• NA	• IVIg
**Katada et al. (2022) **[14]	44/F	Dysmenorrhea, insomnia, palmoplantar pustulosis, umbilical hernia operation, food allergy	Pfizer/second	• Ascending bilateral weakness of the arms • Distal paresthesia of four limbs • Generalized areflexia	1	• Protein: 129 mg/dL • Cells: 1/mm^3^	• Demyelinating sensorimotor polyneuropathy of median, ulnar, and sural nerve • Prolonged/absent F wave latency	• Normal spinal MRI	• Twice IVIg followed by maintaining IVIg therapy
**Devaraj et al. (2022) **[15]	30/M	NA	Covishield (ChAdOx1 nCoV-19)/first	• Quadriparesis • Facial palsy • Truncal ataxia • Generalized areflexia • Impaired posterior column sensation • Bilateral mute plantar response	15	• Albumin-cytological dissociation	• Sensorimotor demyelinating neuropathy with conduction block	• Brain and spinal MRI: enhancement of cauda equina, bilateral trigeminal nerves, and meatal segment of facial nerves	• IVIg for 3 months • IVMP followed by oral steroids
**Singh et al. (2022) **[16]	66/F	Type 2 DM with neuropathy, hypertension, hyperlipidemia	Moderna/ second	• Progressive lower extremity weakness • Difficulty walking • Numbness in bilateral upper extremities • Weight loss	90	• Protein: 237 mg/dL • Cells: 2/mm^3^	• Absent sural sensory nerve response • Prolonged distal latencies • Absent/prolonged F wave • Denervation in all muscles tested in lower limbs	• Normal brain and spinal MRI	• Initial IVIg followed by maintaining IVIg therapy every 4 weeks
**Leemans et al. (2022) **[17]	79/M	NA	Pfizer/first	• Progressive upper and lower extremity weakness • Reduced vibration sense • Global areflexia	2	• Protein: 110 mg/dL • WBC: 6/µL	• Subacute demyelinating neuropathy • Prolonged/absent F-waves • Absent SNAPs	• NA	• IVIg • Methylprednisolone • Azathioprine
	62/M	NA	AstraZeneca/ first	• Paresthesia in the limbs, orally and in the genital area • Reduced sensation to touch and vibration • Positive Romberg sign • Weak reflexes	28	• Not performed	• Demyelinating neuropathy • Prolonged F-waves • Absent SNAPs	• NA	• Oral methylprednisolone with some effect on sensory complaints (stopped early due to intolerance)
**Coelho et al. (2022) **[18]	48/M	Essential arterial hypertension	AstraZeneca/ first and second	• Anosmia, ageusia • Lower limb weakness • After second dose: • Lower limb numbness • Gait instability • Urinary dysfunction • Distal proprioception defect • Mixed lower limb ataxia • Weakened lower limb reflexes	21: after first dose 5: after second dose	• Protein: 247 mg/dL • Cells: 0.8/mm^3^	• Demyelinating sensorimotor polyneuropathy • Increased F-wave latencies	• Spinal MRI: multiple small cervical, thoracic and lumbar T2 hyperintense lesions, without contrast enhancement • Brain MRI: unremarkable	• IVMP followed by oral prednisolone
**Fofiadou et al. (2022) **[19]	62/M	NA	Ad26.COV2.S/ first	• Mild symmetric lower limb weakness • Dysarthria • Facial diplegia • Acral paresthesia • Sensory loss • Absent achilles tendon reflex	18	• Protein: 64 mg/dL • Cells: 0/mm^3^	• Severe bilateral neuropathy with acute and chronic denervation changes	• NA	• IVIg • Plasmapheresis • Pulsed corticosteroid therapy with oral dexamethasone
**Kim et al. (2023**) [20]	72/M	NA	mRNA-1273 vaccine/NA	• Recurrent symmetric distal limb weakness • Sensory dysfunction • Areflexia	30	• Protein: 72 mg/dL • WBC < 5/mm^3^	• Demyelinating polyneuropathy	• NA	• IVIg • Oral prednisolone • Azathioprine
	50/M	Hypertension	Ad26.COV2.S/ first	• Tingling sensation in legs • Gait disturbance • Vibration sensation deficits • Distal lower limb weakness • Areflexia • Positive Romberg test	35	• Protein: 158 mg/dL • WBC < 5/mm^3^	• Motor demyelinating polyneuropathy • Normal NCS of sensory nerves	• No specific findings on MRI of the brain, whole spinal cord, and PET	• High-dose oral prednisolone • Azathioprine • IVIg
**Bendi et al. (2023) **[21]	67/M	NA	Ad26.COV2.S/ NA	• Progressive lower limb weakness • Left facial palsy • Generalized areflexia • Lower extremity ataxia	21	• Albuminocytologic dissociation	• Impersistent F-waves • Decreased recruitment in lower extremities • Demyelination	• NA	• IVIg
**Duncan et al. (2023) **[22]	39/M	None	Pfizer/first	• Bilateral distal paresthesia • Muscle weakness • Fine motor difficulties • Ataxic gait • Areflexia in lower extremities	14	• Increased protein without pleocytosis	• Demyelination	• Normal brain and spinal MRI	• IVIg • Long-term prednisone therapy
**Dennis et al. (2023) **[23]	26/M	NA	Pfizer-BioNTech/first and second	• Bifacial weakness and numbness • Symmetric distally predominant weakness • Paresthesia • Facial diplegia, foot drop, areflexia	41: after first dose 21: after second dose	• Protein: 485 mg/dL • Cells: 4/mm^3^	• Chronic and active demyelinating polyradiculoneuropathy	• Spinal MRI: cauda equina nerve root enhancement	• IVIg
	45/M	NA	Johnson & Johnson/single	• Distal paresthesia and temperature sensitivity in hands and feet • Facial diplegia • Dysarthria • Dysesthesia in feet • Areflexia in left ankle	8	• Protein: 186 mg/dL • Cells: 6/mm^3^	• Diffuse chronic and minimally active demyelinating polyradiculoneuropathy	• Brain MRI: Normal at symptom onset, trigeminal enhancement (4 months later) • Cervical and thoracic spine MRI: unremarkable	• IVIg
	64/F	Mild COVID-19 infection (2 months prior to vaccination)	Pfizer-BioNTech/first	• Bifacial weakness • Dysarthria • Dysphagia • Acroparesthesia • Gait imbalance • Areflexia and sensory loss in lower extremities	8	• Protein: 217 mg/dL • Cells: 6/mm^3^	• Diffuse chronic and active demyelinating polyradiculoneuropathy	• Brain MRI: mild asymmetric enhancement of the left facial nerve	• IVIg
**Kubota et al. (2023) **[24]	39/F	Plasmacytoma	Pfizer/second	• Numbness in legs • Muscle weakness in both hands • Paresthesia and dysesthesia below the knee • Difficulty standing and walking • Areflexia	7	• Protein: 189 mg/dL • Cells: 1/mm^3^	• Abnormal temporal dispersion and reduced motor conduction velocity in right median nerve • Decreased SNAP amplitude and velocity in median and ulnar nerves	• Brain MRI: increased signal of FLAIR in deep white matter • Lumber MRI: swollen cauda equina, enhancement of STIR in the nerve root	• IVIg • Twice IVMP • Oral prednisolone • Plasmapheresis
**Cheng et al. (2023) **[25]	74/M	Osteoarthritis of both knee joints	mRNA-1273/ second	• Weakness of hands and legs • Quadriparesis • Paresthesia over bilateral C8-T1 dermatomes • Impaired vibration and joint position in lower limbs • Generalized areflexia	2–3	• NA	• Typical acquired demyelination • Axonal degeneration • Absent sensory action potentials of sural nerves	• Spinal MRI: thecal sac compression at C4-C5, spondylolisthesis at L4-L5	• Methylprednisolone (continued as intermittent pulse steroid therapy)
**Samakoush et al. (2023) **[8]	67/F	COIVID-19 infection	NA	• Weakness in all extremities • Sensory disturbances • Hyporeflexia	NA	• NA	• Conduction slowing velocity • Axonal sensory and motor polyneuropathy	• NA	• IVIg daily for 5 days; then monthly
**Freir et al. (2023) **[26]	61/M	Hypertension, type-2 diabetes, stroke, chronic lower back pain	AstraZeneca/ first	• Bilateral upper and lower limb weakness • Thoracic back pain • Areflexia	12	• Protein: 191 mg/dL • WBC: 0/mm^3^	• Demyelinating peripheral sensorimotor neuropathy	• Brain and spinal MRI: no causative lesion	• IVIg • Plasma exchange • IVMP followed by oral steroids and azathioprine
**Karbasforooshan et al. (2024)** [27]	NA	NA	Sputnik V/NA	• Progressive flaccid tetraparesis • Dysautonomia	14	• NA	• NA	• NA	• IVIg
**Smaoui et al. (2024) ** [28]	41/M	None	AstraZeneca/ first	• Distal dominant quadriparesis • Four limb paresthesia • Areflexia • Proprioceptive ataxia	15	• Protein: 4.9 g/L • WBC: 1/mm^3^	• Sensorimotor demyelinating polyneuropathy	• Lumbar spine MRI: no sign of myelopathy	• Plasma exchange • IVIg • Oral prednisolone
**Li et al. (2024) ** [29]	42/M	Charcot-MarieTooth neuropathy type 1A	Inactivated vaccine/second	• Distal muscle weakness in all extremities • Paresthesia in distal part of all extremities • Decreased tendon reflexes • Proprioceptive ataxia	7	• Protein: 0.961 g/L • Cells: 1/mm^3^	• Multiple peripheral nerve damage involving demyelination of motor and sensory nerves with axonal damage	• Brachial MRI: swelling of bilateral brachial plexus without marked enhancement of the nerve ganglion • Head and cervical MRI: unremarkable	• IVIg • Oral mattemycophenol ester • Oral prednisone • IVMP (After relapse) • Oral mycofenolate mofetil (After relapse)
**Saito et al. (2024)**[30]	48/F	NA	mRNA-1273/fourth (Pfizer-BioNTech for previous doses)	• Diplopia• Lower extremity weakness• Absent lower limb reflexes• Sensory disturbances	2	• Protein: 75 mg/dL• WBC: 2.3/μL	• Demyelinating activity in bilateral tibial nerve• Prolonged minimum latency	• NA	• Steroid pulse therapy• IVIg
**Goldberg et al. (2024) **[31]	60/M	None	NA	• Progressive bilateral lower extremity weakness • Respiratory failure • Generalized areflexia • Sensory deficits	14	• Albuminocytologic dissociation	• Demyelinating polyneuropathy	• Normal brain MRI	• IVIg • Steroid therapy
**Bahramy et al. (our case)**	67/M	DM, knee arthrosis	Sinopharm/ third	• Progressive paresthesia and weakness in all extremities • Generalized areflexia • Ataxia	7	• Protein: 210 mg/dL • WBC: 4/mm^3^	• Absent CMAP amplitude in lower limbs • Prolonged distal latency, slowed conduction velocity • Demyelinating polyneuropathy	• Cervical MRI: normal • Lumbosacral MRI: bulging of the L5-S1 disc	• IVIg • Azathioprine • Prednisolone • Rehabilitation

*Abbreviations: CIDP* Chronic inflammatory demyelinating polyneuropathy, *CMAP* Compound muscle action potentials, *CSF* Cerebrospinal fluid, *DM* Diabetes mellitus, *EMG-NCV *Electromyography and nerve conduction studies, *F* Female, *FLAIR* Fluid attenuated inversion recovery, *IVIg* Intravenous immunoglobulin, *IVMP* Intravenous methylprednisolone, *M* Male, *MRI* Magnetic resonance imaging, *NA* Not available, *PET* Positron emission tomography-computed tomography, *SNAP* Sensory nerve action potential, *STIR* Short tau inversion recovery, *WBC* white blood cell count

Due to the vast number of vaccinated people, some of the neurological conditions will manifest within the post-vaccination period. Singh et al. reported a 66-year-old female with progressive lower extremity weakness after receiving the Moderna COVID-19 vaccine three months prior to the onset of symptoms. A close temporal link between the onset of symptoms and vaccination contributed to identifying the causality of this adverse event following immunization. It is critical to remember that, as with any other vaccination, any suggested link between COVID-19 vaccination and demyelinating neuropathies cannot be considered causal based on the limited case reports relative to the number of people immunized. However, the unfounded link between these two should not prevent further vaccination; rather, it should increase awareness regarding post-vaccination assessments [16, 23].

As demonstrated in Table 4, a total of 32 cases of CIDP following COVID-19 vaccination have been reported till July 2024, among which only three patients had received inactivated vaccine. Meanwhile, ten patients presented with peripheral neuropathy after mRNA vaccines, and seventeen developed such symptoms following adenovirus vector vaccines. A review by Hosseini and Askari reported that Pfizer, Moderna, AstraZeneca, and Johnson & Johnson vaccines were associated with neurological complications more than Sinopharm and other inactivated vaccines [32]. This association hints towards the fact that such adverse events are less likely following inactivated vaccines. It also emphasizes the importance of considering them in the differential diagnosis while choosing the most appropriate vaccine based on the patient’s comorbid disease and history.

Moreover, most of the reviewed patients presented with demyelinating symptoms after the first or second dose of vaccination and within 14–21 days after the administration. In contrast, our presented case developed such symptoms a week after the third dose of an inactivated vaccine, which highlights the rarity and importance of this report.

CIDP is often considered to be the chronic counterpart of acute inflammatory demyelinating polyradiculoneuropathy (AIDP), the most common form of GBS. Around 16% of CIDP patients may present acutely mimicking GBS but are then followed by a chronic course lasting more than eight weeks [10]. Also, Luca et al. presented an uncommon variant of post-vaccination CIDP known as chronic inflammatory axonal polyneuropathy [33]. The challenge of determining whether a patient has CIDP is greatest in patients with diabetes [34]. In our case, the patient had a medical history of diabetes, which led to thinking of his first symptoms as signs of diabetic neuropathy (DPN). Similarly, Wang et al. described a case of CIDP in a young female, simulating DPN [35]. Given the overlap of clinical presentations between CIDP, DPN, and other demyelinating neuropathies, it is essential to distinguish CIDP from the mentioned conditions because misdiagnosis may lead to delayed therapy and a terrible prognosis.

Typical CIDP is characterized by symmetrical weakness in proximal and distal muscles that develops progressively over eight weeks or longer [36]. As appreciated in our case, and according to the cases mentioned in Table 4, 26 patients presented with progressive lower extremity paresthesia, weakness, or both. Also, almost all cases documented diminished or absent tendon reflexes and gait ataxia. Acknowledgment of these presenting signs and symptoms will further help physicians in the timely diagnosis of this condition.

Laboratory tests are crucial in eliminating other causes of peripheral neuropathy. Recent reports have shown that lab tests are usually within normal limits [37]. On CSF analysis, elevated protein concentration is a common finding similar to our case. Around 90% of patients with CIDP have a high CSF protein level without pleocytosis [10, 16]. As evidenced in many studies, spinal MRI, as the main imaging modality, can support the diagnosis of CIDP by showing contrast enhancement of the cauda equina or lumbosacral nerve roots. MRI results of our patient were unremarkable except for a mild herniated intervertebral disc. However, the patient’s spinal MRI did not have findings consistent with any other neurological condition [16, 22, 37]. EMG-NCV findings of our case were aligned with that of many other studies revealing primary demyelination of peripheral nerves, which is compatible with CIDP [20, 22, 35]. CSF analysis, electrophysiological studies, and MRI results are important diagnostics for excluding other possible causes. Of note, the absence of electrophysiological or histological evidence consistent with CIDP does not preclude the diagnosis, and it is essential to look at the diagnostic panel as a whole.

Just like our case, the management of CIDP patients is divided into pharmacological and non-pharmacological interventions. The central axis of non-pharmacological management is rehabilitation [38]. As supported by the recent literature, IVIg, prednisolone, and plasmapheresis are the recognized standard treatments for CIDP, either alone or in combination. Azathioprine, cyclophosphamide, cyclosporin A, and interferon-a are a few of the additional medications that are helpful for patients who did not respond to the above-mentioned treatment options [36, 37, 39]. Proper response to first-line treatment options is warranted by the early diagnosis of this condition.

In conclusion, CIDP should be considered a rare complication following the COVID-19 vaccination. While the benefits of immunization outweigh the risks, healthcare providers should be aware of this potential complication. Our review of the literature adds to the limited evidence, implying the role of vaccination in the pathogenesis of CIDP, and points out the importance and rarity of these conditions following inactivated vaccines. This report also provides one of the first detailed descriptions of chronic inflammatory neuropathies triggered by the Sinopharm vaccine.

### Supplementary Information

Supplementary Material 1.

## Data Availability

All data regarding the case have been reported in the manuscript. Kindly contact the corresponding author in case of requiring any further information.

## Abbreviations

AIDP: Acute inflammatory demyelinating polyradiculoneuropathy
ANCA: Antineutrophil cytoplasmic antibody
CIDP: Chronic inflammatory demyelinating polyneuropathy
CMAP: Compound muscle action potentials
COVID-19: Coronavirus disease 2019
CSF: Cerebrospinal fluid
DPN: Diabetic neuropathy
EMG-NCV: Electromyography and nerve conduction studies
GBS: Guillain-Barré syndrome
IVIg: Intravenous immunoglobulin
MRC: Medical Research Council
MRI: Magnetic Resonance Imaging
PMN: Polymorphonuclear neutrophil
SNAP: Sensory nerve action potential
WBC: White blood cell
