# Supplementary material for: Chronic inflammatory demyelinating polyneuropathy following COVID-19 vaccination: a case report and literature review
Source: BMC Neurol. 2024 Jul 29;24:262. doi: 10.1186/s12883-024-03756-3 (PMC11285274; doi:10.1186/s12883-024-03756-3)
Supplement: Supplementary file 1 — Supplementary Material 1. [file 12883_2024_3756_MOESM1_ESM.pdf]

CARE Checklist of information to include when writing a case report

CARE case report guidelines

(cc) BY-NC-ND

Topic	Item	Checklist item description	Reported on Line
Title	1	The diagnosis or intervention of primary focus followed by the words "case report"	1-2
Key Words	2	2 to 5 key words that identify diagnoses or interventions in this case report, including "case report"	28-29
Abstract (no references)	3a	Introduction: What is unique about this case and what does it add to the scientific literature?	11-15
	3b	Main symptoms and/or important clinical findings	16-18
	3c	The main diagnoses, therapeutic interventions, and outcomes	20-24
	3d	Conclusion—What is the main "take-away" lesson(s) from this case?	25-27
Introduction	4	One or two paragraphs summarizing why this case is unique (**may include references**)	40-52
Patient Information	5a	De-identified patient specific information.	55
	5b	Primary concerns and symptoms of the patient.	55-57,60-63
	5c	Medical, family, and psycho-social history including relevant genetic information	57-59
	5d	Relevant past interventions with outcomes	63-68
Clinical Findings	6	Describe significant physical examination (PE) and important clinical findings.	69-77
Timeline	7	Historical and current information from this episode of care organized as a timeline	56-68, 78-87,94-98, 110-127
Diagnostic Assessment	8a	Diagnostic testing (such as PE, laboratory testing, imaging, surveys).	63-64, 78-85, 94-98, 103-109
	8b	Diagnostic challenges (such as access to testing, financial, or cultural)	NA
	8c	Diagnosis (including other diagnoses considered)	110-112
	8d	Prognosis (such as staging in oncology) where applicable	NA
Therapeutic Intervention	9a	Types of therapeutic intervention (such as pharmacologic, surgical, preventive, self-care)	112-117
	9b	Administration of therapeutic intervention (such as dosage, strength, duration)	112-117
	9c	Changes in therapeutic intervention (with rationale)	115-117
Follow-up and Outcomes	10a	Clinician and patient-assessed outcomes (if available)	118-127
	10b	Important follow-up diagnostic and other test results	120-127
	10c	Intervention adherence and tolerability (How was this assessed?)	118-127
	10d	Adverse and unanticipated events	NA
Discussion	11a	A scientific discussion of the strengths AND limitations associated with this case report	138-157, 159-162
	11b	Discussion of the relevant medical literature **with references**.	129-202
	11c	The scientific rationale for any conclusions (including assessment of possible causes)	129-147, 163-174
	11d	The primary "take-away" lessons of this case report (without references) in a one paragraph conclusion	203-209
Patient Perspective	12	The patient should share their perspective in one to two paragraphs on the treatment(s) they received	118-120
Informed Consent	13	Did the patient give informed consent? Please provide if requested	Yes ☑ No ☐
